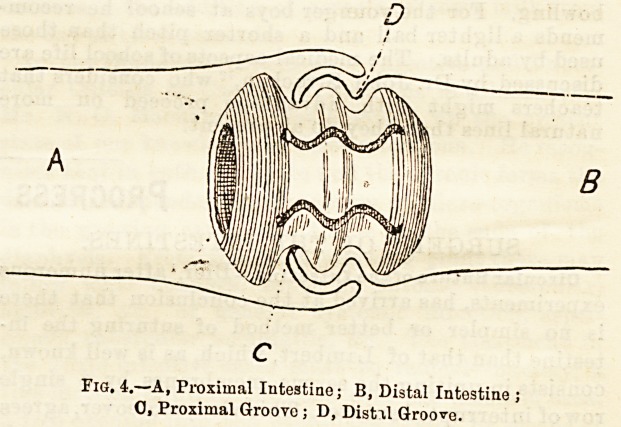# Progress in Surgery

**Published:** 1896-04-04

**Authors:** 


### PROGRESS IN SURGERY.

### SURGERY OF THE INTESTINES.

**Circular Suture of the Intestine.**—Bier,[1] after numerous experiments, has arrived at the conclusion that there is no simpler or better method of suturing the intestine than that of Lambert, which, as is well known, consists in uniting the serous membranes by a single row of interrupted sutures. This view, moreover, agrees with that of a number of distinguished surgeons, such as Hahn, von Bergmann, Riedel, and Trendelenburg, though many others have adopted the double suture, described as the Czerny-Lembert method. Dr. Bier has on fifteen occasions adopted the Lembert suture for resection of the intestine. Of these two died, but in neither case was death to be attributed to the method of suturing employed. The operation can be finished in from fifteen to twenty-five minutes. It is, therefore, useless to have recourse to more complicated methods, which the author severely criticises, the inconvenience of Senn's plates and Murphy's button in his opinion not being compensated by any real advantage.

**Intestinal Resection.**—Allingham[2] has operated on ten cases, of which six recovered and four died. Distension of the intestined at the time of resection enormously increases the risk of the operation. The explanation of this circumstance is probably that large quantities of fæces pressed down towards the site of union, and constantly disturbed it by the peristaltic waves they set up, and the patient is, so to speak, poisoned by the fæcal accumulation, thus hindering union of the divided ends of intestine. In large intestine cases fæces may block the bobbin, and even tear it away. It follows that large intestines must not be resected when the gut above the stricture is distended by fæces. A colotomy should be performed, by which means the intestine can be thoroughly emptied, and resection and anastomosis should be performed subsequently. Stricture of the small intestine may be resected even when distended, because the fæces are usually liquid.

**New Bobbins for Intestinal Anastomosis.**—To obviate many of the disadvantages of the ordinary bobbins Allingham[3] has devised a new one, the principle of which rests in the fact that it consists of two cones with their apices coming together to form the centre.

FIG. 1.

The tubes are of bone or ivory, the shape of two hollow truncated cones with their lesser ends together, having the appearance of small dice-boxes. These are carefully decalcified to within about three-sixteenths of the centre (*a*), leaving at the junction of the two cones a hard, unyielding portion upon which any pressure from the sutures is borne. The ends of the tube are quite soft.

This appliance serves all the purposes required of a button or bobbin, for it keeps the parts at absolute rest; can be absorbed, as it is made of decalcified bone; and can be liberated without any absorption or sloughing of the parts it unites. Moreover, from its shape it cannot, until the decalcified bone is absorbed, slip away from the seat of union, and when the sutures in each piece of intestine are tied, the parts to be joined are brought together, and yet no excessive pressure is exerted on the edges which it is desired to connect.

Fig. 2.

As the bobbin may be used in various operations, it has been made in different sizes. In the union of divided intestine a running "purse-string" stitch is taken round each end of the gut, the bobbin inserted, and as the stitches are tightened the parts are brought down to the centre of the bobbin, for, as the bobbin consists of two cones with their apices pointing to its centre, the tighter the sutures are drawn the more completely must the intestine be drawn to the apex of the cone which has been inserted into it.

A few Lembert's sutures may then be inserted at various points, or a continuous Lembert's suture may be used all round if that be thought necessary. It is wise to scarify the peritoneum of the intestines with a needle for about half an inch around the seat of union to promote the exudation of lymph, especially if no Lembert's sutures have been employed. Allingham has used this bobbin once on the human subject with excellent results. Another ingenious bone or ivory bobbin has been devised by Hayes[4] of the form here depicted. (Fig. 3.)

It will be seen that the central part has been longitudinally segmented by means of a fine saw, the

Fig. 3.

number of sections being proportionate to the size of the bobbin. The ends of the bobbin, up to and a little beyond the extremities of the section lines, are decalcified. End-to-end union—as after an enterectomy—is to be accomplished in the following manner. At the orifice of each portion of intestine a running marginal suture traversing all layers of the gut wall is to be inserted. On the proximal part of the intestine, about three-quarters of an inch above the marginal suture, a purse-string suture, implicating the serous coat only, is to be introduced. The ends of intestine, proximal and distal, are now to be drawn over the bobbin, until they correspond to the upper, or proximal groove marked A in Fig. 3. Their marginal sutures are then to be drawn tight so as to make them fit well into it, and securely tied. The final step consists in gently pushing up the bobbin and distal intestine, whilst careful downward traction is being made on the proximal intestine, until invagination from below upwards will have proved sufficient to bring the sub-serous purse-string on the proximal intestine over the site of the bobbin groove B (Fig. 3). This purse-string, on being tied, will press the serous surfaces of the proximal and distal ends of intestine together, and by lightly compressing the distal portion over the corresponding groove of the bobbin will afford absolute security against the risk of slipping (Fig. 4.)

Special features of the bobbin are: 1. Its undecalcified condition, where strength is required to bear pressure of ligature-like sutures. 2. Its decalcified condition at the extremities, where solution takes place within a moderate period. 3. The undecalcified segmented centre, which must fall as small fragments into the lumen of the intestine, and are therefore unlikely to cause any obstruction. A somewhat similar

Fig. 4.—A, Proximal Intestine; B, Distal Intestine; C, Proximal Groove; D, Distal Groove.

bobbin, but having one groove instead of two, can be used for lateral anastomosis by a slight modification of the end-to-end method of junction just described.

**Maunsell's Method of Intestinal Anastomosis** is claimed by Wiggin[5] to be one of the best means of effecting this operation, and he draws attention to the importance of the following points in the technique: 1. The longitudinal slit which is made in the segment of bowel having the greatest calibre (proximal or distal), and through which the invagination occurs) should be located at least two inches from the cut end of the bowel. 2. The mesentery of both segments must be included in the first temporary suture which is passed at this intestinal border; this prevents sloughing of the bowel at this point. 3. The sutures should be placed at least a quarter of an inch from the cut intestinal edge; they should be interrupted, about twenty in number, and should not be drawn too tightly when they are tied. 4. The best suture material for this work is carefully tested and prepared horsehair. 5. The needle best adapted to this work is a round straight one (milliner's, Nos. 6 to 9). 6. The invagination after the sutures have been placed must be carefully reduced, rather by manipulation than by traction, otherwise the sutures may cut out. 7. In closing the longitudinal slit, too much of the intestinal edges should not be turned in, or a contraction may result at this point. The special claim of this method of intestinal suture rests upon the fact that no special appliance is needed; its adaptability to every portion of the intestinal tract; the ease, rapidity, and safety of the operation; and the fact that no time need be lost in determining the direction in which the invagination should be made. Ullman,[6] who also advocates this method warmly, thinks that some may hesitate to adopt it because one simple row of sutures is used; others because the sutures include all three of the coats of the intestine. To the first it may be said that a second series of sutures may be applied to the serosa after the gut has been drawn out, but this he considers unnecessary. As to the second objection, he claims that it makes no difference whether the mucosa is included in the suture or not. If the surgeon does not wish to include it, he can pierce only the muscularis and serosa, for the field of operation is so plainly in view and under control that the mucosa can easily be omitted from the suture. Rose[7] also reports a successful case by this method, and thinks that chromicised catgut is the material best adapted for the stitches.

**Murphy's Button.**—The value of this contrivance has been made the subject of experimental inquiry by Marwedel,[8] who regards it as superior to all other mechanical devices for doing away with suture. It is simple, and the operation can be done in a very short time. On the other hand, it has serious drawbacks, which will prevent its general adoption. On the whole, it can only be recommended in cases in which the patient's condition is such as to necessitate a speedy termination of the operation, as in severe traumatic lesions of the abdomen, in certain cases of ileus and gangrene of the intestine, &c. Chaput[9] thinks the use of Murphy's button is attended by a great many inconveniences in certain cases. It does not always shorten the duration of the operation, provided all necessary precautions be taken; in addition, it is more liable to result in perforation than continuous suturing in three rows, an operation which takes but a few minutes more to perform in the hands of an experienced surgeon. When the intestine is empty, suturing appears to be preferable to Murphy's method. The use of the button is formally contra-indicated when the walls of the stomach or intestine are too thick; it is indicated principally in cases of obstruction or gangrenous hernia, and whenever it is necessary to finish up rapidly an operation which has already lasted too long.

**Intussusception.**—Rydygier,[10] from a study of eighty-four cases, concludes as follows: In acute intestinal invagination—1. Operation should be resorted to as early as possible after non-operative methods have been thoroughly tried without success. 2. After a laparotomy disinvagination is of value when it can be accomplished without any great difficulty. If the intestinal wall is suspicious-looking at the point of invagination, the peritoneal cavity should be walled off with iodoform gauze, or the particular portion of intestine should be drawn outside the abdominal wall. 3. When disinvagination is impossible, the resection of the invagination is the least dangerous procedure. 4. The resection of the entire invagination should be performed when the invaginating sheath is so markedly altered that there is danger of perforation. 5. The formation of an artificial anus or an entero-anastomosis have usually no place in these cases; only in the presence of collapse is the formation of an artificial anus permissible. In chronic invagination internal medication and non-operative methods must be employed, but should be abandoned after a week's trial. Disinvagination is of little avail, although four successful cases have been reported. Resection of the invagination is here also the operative method of choice. Entero-anastomosis should only be undertaken in the presence of adhesions. An artificial anus should not be thought of in the treatment of chronic invagination. Eve[11] records a successful case of laparotomy for intussusception in an infant, and draws attention to the rise of temperature which appears to be a common accompaniment of these cases, and which, therefore, should be anticipated by cold sponging, &c.

**Simple Stricture of the Intestine** is very rare. A case is reported by Robson,[12] in which he modified the ordinary enteroplasty performed by Heineke and Mikulicz by the use of a bone bobbin. The advantages of this method are: (1) Rapidity of execution, since only two continuous sutures are required—one to unite the mucous margin, the other the serous; (2) an immediately patent and efficient channel, preventing tension above the newly-formed gut; (3) protection to the line of sutures until the lymph has become partly organized.

**Intestinal Obstruction.**—Cartledge[13] thinks that the large death-rate from operation in acute intestinal obstruction does not come from lack of adequate surgical methods, but from the late time at which the relief is supplied. Barton[14] considers that in acute obstruction the patient is never in a condition to have the shortest anastomosis operation performed at the primary operation. If the strangulated gut be gangrenous it should be removed, and an artificial anus made; an anastomosis operation may be performed later if the patient survive. Interesting cases are recorded by Griffiths,[15] Pick,[16] and Murray.[17]

**Colotomy** is recommended by Wheeler[18] prior to excision of the rectum for cancerous disease in cases where the subject is suffering from much rectal irritation, diarrhœa, pain, and consequent nervous exhaustion, in order that the irritated bowel might get rest and also improve the constitutional powers. White and Golding-Bird[19] record a case in which colotomy was performed for membranous colitis. For five weeks all the motions were passed through the artificial anus without purgatives. As at the end of this time all the original symptoms had disappeared, the colotomy wound was closed. Two months afterwards the patient was feeling better than she had done for years. The authors submit that in a severe case of membranous colitis this treatment is justifiable and most rational, for by it the colon has complete rest.

**Colectomy** is best effected, says Robson,[20] by the aid of the decalcified bone bobbin. He records five cases, some of which were for the removal of a tumour, and some for the cure of fæcal fistulæ in which either a bone bobbin or Murphy's button is used. The records at the Leeds Infirmary (13 cases) during the past two years show a mortality of 23 per cent.

**Volvulus of the Sigmoid** in a man of 85 years, successfully treated by cœliotomy, is reported by Smith and Flemming.[21] Great stress is laid on the beneficial effect of draining the bowels by the insertion of a rubber tube.

[1] Med. Week, Oct. 18th, 1895; and Arch. f. Klin. Chir. xlix, 4. [2] Brit. Med. Journ., Nov. 2nd, 1895. [3] Lancet, Aug. 31st, 1895, p. 518. [4] Ibidem, Dec. 28th, 1895, p. 1,619. [5] New York Med. Journ., Dec. 14th, 1895. [6] Annals of Surgery, Aug. 1895; and Cent. fur Chir., No. 2, 1895. [7] Practitioner, Aug., 1895. [8] Med. Week, Oct. 25th, 1895; and Beitr. z. Klin. Chir., xiii., 3. [9] Med. Week, Aug. 2nd, 1895. [10] Amer. Journ. Med. Sci., Nov. 1895: and Beit. z. Centr. f. Chir., 1895, No. 27. [11] Brit. Med. Journ., Oct. 10th, 1895, p. 968. [12] Lancet, Aug. 3rd, 1895, p. 258. [13] Med. Chron., July 1895. [14] Therapeut. Gazette, Sept. 16th, 1895. [15] Lancet, May 25th, 1895. [16] Clinic. Journ., June 12th, 1895. [17] New York Med. Rec., Oct. 19th, 1895. [18] Brit. Med. Journ., Nov. 30th, 1895. [19] Ibidem, Dec. 21st, 1895, p. 1,559. [20] Ibidem, Oct. 19th, 1895, p. 963. [21] Ibidem, July 20th, 1895.

## Figures and Tables

**Fig. 1. f1:**
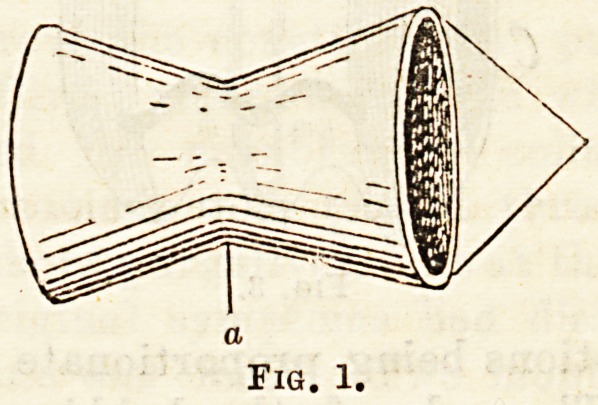


**Fig. 2. f2:**
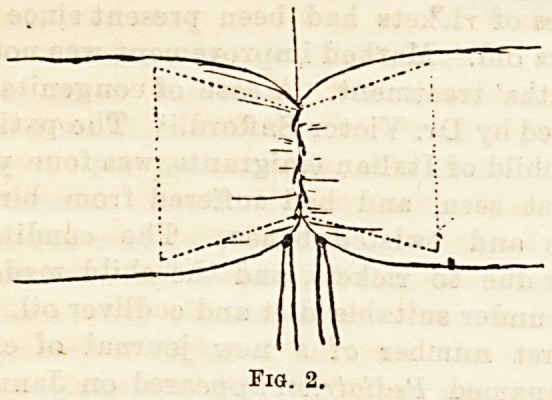


**Fig. 3. f3:**
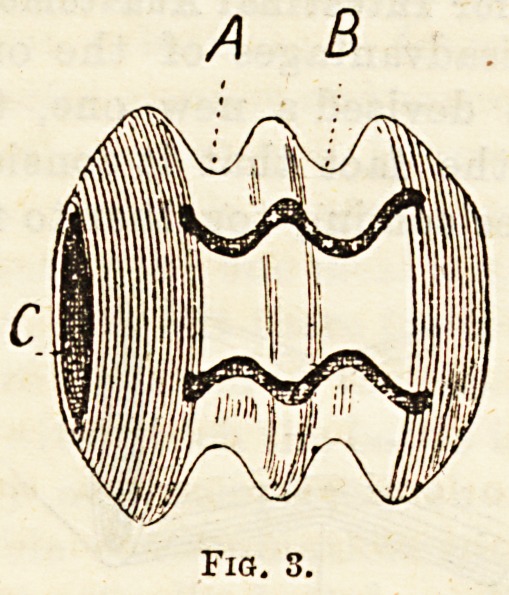


**Fig. 4. f4:**